# Tuning attention based long-short term memory neural networks for Parkinson’s disease detection using modified metaheuristics

**DOI:** 10.1038/s41598-024-54680-y

**Published:** 2024-02-21

**Authors:** Aleksa Cuk, Timea Bezdan, Luka Jovanovic, Milos Antonijevic, Milos Stankovic, Vladimir Simic, Miodrag Zivkovic, Nebojsa Bacanin

**Affiliations:** 1 Present Address: Singidunum University, Danijelova 32, Belgrade, 11010 Serbia; 2 Faculty of Transport and Traffic Engineering, University of Belgrade, Vojvode Stepe 305, Belgrade, 11010 Serbia; 3 College of Engineering, Department of Industrial Engineering and Management, Yuan Ze University, Taoyuan City, 320315 Taiwan; 4 College of Informatics, Korea University, 145, Anam-ro, Seongbuk-gu, Seoul, Republic of Korea; 5 MEU Research Unit, Middle East University, Amman, Jordan; 6 Faculty of Data Science and Information Technology, INTI International University, 71800 Nilai, Malaysia

**Keywords:** Parkinson’s disease, Medical diagnosis, Long-short term memory neural networks, Crayfish optimization algorithm, Optimization, Computational models, Computational platforms and environments, Data mining, Machine learning

## Abstract

Parkinson’s disease (PD) is a progressively debilitating neurodegenerative disorder that primarily affects the dopaminergic system in the basal ganglia, impacting millions of individuals globally. The clinical manifestations of the disease include resting tremors, muscle rigidity, bradykinesia, and postural instability. Diagnosis relies mainly on clinical evaluation, lacking reliable diagnostic tests and being inherently imprecise and subjective. Early detection of PD is crucial for initiating treatments that, while unable to cure the chronic condition, can enhance the life quality of patients and alleviate symptoms. This study explores the potential of utilizing long-short term memory neural networks (LSTM) with attention mechanisms to detect Parkinson’s disease based on dual-task walking test data. Given that the performance of networks is significantly inductance by architecture and training parameter choices, a modified version of the recently introduced crayfish optimization algorithm (COA) is proposed, specifically tailored to the requirements of this investigation. The proposed optimizer is assessed on a publicly accessible real-world clinical gait in Parkinson’s disease dataset, and the results demonstrate its promise, achieving an accuracy of 87.4187 % for the best-constructed models.

## Introduction

Parkinson’s disease (PD) is a progressively debilitating neurodegenerative disorder primarily affecting the dopaminergic system in the basal ganglia, and it impacts millions of individuals worldwide^1^. The initial description of PD was provided by James Parkinson in his essay “An Essay on the Shaking Palsy”^2^, published in 1817. This essay is often regarded as groundwork for Parkinson’s disease understanding. James Parkinson’s essay described several key clinical characteristics of the disease, encompassing resting tremors, muscle rigidity, bradykinesia (slowness of movement), and postural instability. He referred to the condition as “shaking palsy,” emphasizing the tremors observed in the individuals affected by the disease.

Currently, PD diagnosis primarily relies on clinical evaluation without reliable diagnostic tests^3^, and it can be imprecise and inherently subjective. Standardized rating tools, such as the Unified Parkinson’s Disease Rating Scale (UPDRS), were developed to assess various aspects of PD symptoms. Additionally, the finger-tapping test (FTT), as part of UPDRS, can serve as a clinical marker for evaluating motor performance. FTT is a psychomotor task that involves repetitive tapping movements for evaluating motor function in individuals affected with PD. The FTT involves tapping by the thumb or middle finger as quickly and accurately as possible over a set duration. Variations of the test include unilateral and bilateral tapping, with the latter offering insights into coordination and symmetry. Individuals with PD exhibit impaired finger-tapping performance compared to healthy controls. Key findings in studies include reduced tapping speed (bradykinesia), increased variability in tapping intervals, and reduced tapping amplitude. FTT can be a promising tool for detecting PD in early stages by combining it with other tools and measurements.

Artificial intelligence (AI) has revolutionized healthcare by offering data-driven solutions for complex medical problems. In medical datasets, deep learning methods, have demonstrated exceptional potential in extracting intricate patterns. In healthcare, the application of Long Short-Term Memory (LSTM) networks, a type of recurrent neural network (RNN) is particularly promising for analyzing medical data. Additionally, metaheuristic optimization algorithms have emerged as powerful methods for optimizing the parameters of complex models, enhancing their performance.

An extensive literature review suggests that there is a research gap concerning the application of optimized LSTM networks for Parkinson’s diagnosis. Additionally, the application of emerging optimizers such as the crayfish optimization algorithm (COA)^4^. has yet to be explored. The main focus of this work is to address this issue presenting a novel diagnostic approach for PD by combining the capabilities of deep LSTM networks, optimized by a modified metaheuristic algorithm. The primary goal of this work is to address the need for early and accurate PD diagnosis. An additional scientific contribution of this work is a proposal for a modified version of the COA that tackles some of the observed drawbacks of the original algorithm.

For the experiments, a dataset comprising recordings from inertial wearable sensors with gyroscopes is employed. This dataset encompasses recordings collected from individuals affected by PD, those with atypical PD, and healthy control subjects. During data acquisition, a 3D gyroscope was meticulously positioned inside the patient’s shoe soles, and participants were instructed to walk down a well-lit path while counting backward from 500 in increments of 7, known as a dual-task walk test. Multiple trials were conducted for each participant, and it’s important to note that the data exclusively pertained to the right hand, which was typically the more affected hand in these individuals.

The primary scientific contributions of this work can be outlined as follows:

- A proposal for a novel time-series classification-based approach for PD detection in affected individuals.
- An innovative application of the recently proposed COA for parameter optimization of LSTM tasked by PD diagnosis.
- A modified version of the COA specifically developed for this study and to address the drawbacks of the original algorithm.

## Background and related works

The integration of AI into the realm of medical diagnostics has garnered substantial scholarly interest and is effecting a profound transformation within the healthcare sector. AI presents a promising technique to enhance the accuracy of medical diagnoses, reduce healthcare costs, and improve patient outcomes. AI has been widely applied in radiology to assist in the diagnosis of diseases from X-rays, CT scans, and MRIs. Notable applications include the early detection of lung cancer CT scans using a 3-dimensional deep learning algorithm^5^ and the identification of diabetic retinopathy using networks trained by a dataset of retinal fundus photographs^6^. AI-driven pathology, particularly in the field of digital pathology, has advanced the accuracy of cancer diagnosis and tumor classification. Deep learning models have been employed to aid pathologists in identifying and grading cancers^7^. In cardiology, AI has shown potential in analyzing electrocardiograms (ECGs) for arrhythmia detection^8^ and echocardiograms for cardiac disease assessment. Preceding works have demonstrate impressive results for arrhythmia detection by integrating optimization techniques to tackle large search spaces for parameter optimization^9^ exceeding 98% accuracy.

Machine learning and deep learning methodologies have unequivocally exhibited their efficacy in the field of neurodiagnostics. These sophisticated algorithms are adept at parsing intricate neurophysiological data, encompassing medical imagery, electrophysiological measurements, and behavioral evaluations, thereby culminating in heightened precision and expedience in the diagnostic process. AI-enabled systems are poised to contribute significantly to the timely identification and categorization of neurological disorders, including but not limited to Alzheimer’s disease, multiple sclerosis, and intracranial neoplasms^10,11^. AI has engendered notable enhancements in the scrutiny of electrophysiological data, encompassing electroencephalography (EEG) and magnetoencephalography (MEG) signals, with the express purpose of diagnosing and overseeing conditions such as epilepsy, sleep disorders, and various other neurological maladies. Deep learning algorithms remain essential for the identification of aberrations, the precise localization of epileptic foci, and the prognostication of seizure occurrences. Khan et al. conducted an evaluation, comparing two distinct deep learning methodologies^12^.

The utilization of the finger-tapping test as a diagnostic modality for Parkinson’s disease has garnered attention within the realm of clinical investigation. This test serves as an evaluative measure of the motor function and dexterity of the fingers, presenting itself as a prospective instrument for the early detection and continuous monitoring of Parkinson’s disease. Akram et al^13^. developed a new Distal Finger Tapping (DFT) test to assess distal upper-limb function in PD patients, focusing on kinetic parameters like kinesia score (KS20), akinesia time (AT20), and incoordination score (IS20). The DFT test effectively discriminated between PD patients and controls, with KS20 exhibiting the highest sensitivity (79%) and an area under the receiver operating characteristic curve (AUC) of 0.90. In a research undertaken by Williams et al^14^. a new computer vision technology, DeepLabCut, was used to track and measure finger tapping in smartphone videos to objectively assess bradykinesia in Parkinson’s disease. The computer measures, including tapping speed, amplitude, and rhythm, correlated well with clinical ratings from movement disorder neurologists, demonstrating its accuracy (Spearman coefficients ranged from −  0.50 to −  0.74, $p<$ .001). DeepLabCut offers a ’contactless’ and easily accessible method for quantifying Parkinson’s bradykinesia during clinical examinations, with potential applications in other neurological disorders characterized by altered movements.

Preceding works have tackled PD diagnosis using MRI image analysis reporting outcomes raining form 78%^15^ to 88%^16^. However, the use of MRI is significantly higher d to shoe mounted sensing systems. One major advantage of the proposed approach is the significantly lower diagnosis costs as well as greater availability of diagnosis tools. Researchers also considered handwriting analysis for diagnosis. The paper^17^ tested several classifiers with the best accuracy demonstrated by the Naive Bayes models aching an accuracy of 88.63%. Researchers have considered the use of generative adversarial networks to tackles issues associated with data availability for gait freezing in PS patients^18^. Models trained on the augmented data arrained a reported an exceeding of 90%, however the use of data optimization techniques has not considered in this work. There is an evident research gap for using timeseries PD detection, as well as the application of parameter tuning via metastatic algorithms in the field of PD diagnosis. This work seeks to address the observed gap by proposing a low cost AI powered approach.

### Attention based LSTM

The LSTM^19^ represents a variant of RNNs. These networks retain prior information and incorporate it into their processing of current input data. However, a limitation of traditional RNNs is their inability to effectively capture long-term dependencies, mainly because of the vanishing gradient issue. LSTMs, on the other hand, are purposefully engineered to avoid these challenges associated with long-term dependencies.

The cell state is a crucial component of the LSTM network, which is designed to capture and carry information over long-term dependencies. The hidden state is computed at each time step based on the cell state and the input at that time step. It serves as the output of the LSTM at each step and contains information that the network has learned to be significant for making predictions. The third main element of LSTMs is the gates and they incorporate three different gates for controlling the information flow, the forget gate, the input gate, and the output gate. These gates play an important role in LSTMs to selectively modify and utilize information from the cell state, managing the flow of data within the network. This capability empowers LSTMs to grasp and apply both short-term and long-term dependencies in sequential data.

The forget gate decides which information from the prior cell state should be forgotten. The input gate is responsible for deciding which new information should be incorporated into the cell state. The output gate regulates which information should be extracted from the cell state and utilized in generating the hidden state and output of the LSTM. The LSTM defines the gate, forget gate, cell state, output gate, and hidden state through the following mathematical formulations:

1 $$\begin{aligned} i_t = \sigma (W_{xi}x_t + W_{hi}h_{t-1} + W_{ci}c_{t-1} + b_i) \end{aligned}$$

where $i_t$ refers to input gate activation at time *t*, $x_t$ is the input at time *t*. The hidden state and the cell state at time $t-1$ are referred to by $h_{t-1}$, and $t-1$ respectively. Cell state at time $t-1$ is denoted by $c_{t-1}$. $W_{xi}, W_{hi}, W_{ci}, b_i$ are the weight matrices and bias vectors for the input gate. $\sigma$ denotes the Sigmoid activation function.

2 $$\begin{aligned} f_t = \sigma (W_{xf}x_t + W_{hf}h_{t-1} + W_{cf}c_{t-1} + b_f) \end{aligned}$$

where $f_t$ denotes the forget gate activation at time *t*.

3 $$\begin{aligned} c_t = f_t \cdot c_{t-1} + i_t \cdot \tanh (W_{xc}x_t + W_{hc}h_{t-1} + b_c) \end{aligned}$$

where $c_t$ denotes the cell state at time *t*. $\tanh$ refers to the hyperbolic tangent activation function defined as follows:

4 $$\begin{aligned}{} & {} \tanh (x) = \frac{e^{x} - e^{-x}}{e^{x} + e^{-x}} \end{aligned}$$

5 $$\begin{aligned}{} & {} o_t = \sigma (W_{xo}x_t + W_{ho}h_{t-1} + W_{co}c_t + b_o) \end{aligned}$$

where $o_t$ denotes the output gate activation at time *t*.

6 $$\begin{aligned} h_t = o_t \cdot \tanh (c_t) \end{aligned}$$

where $h_t$ denotes the hidden state at time *t*.

The attention phenomenon lacks a precise mathematical definition, and its incorporation into the Luong attention-based model should be viewed as a mechanism. Networks capable of operating with this attention mechanism and possessing LSTM characteristics are considered attention-based. The primary goal of such a mechanism is to assign varying weights to the input sequence, allowing for the capture of data and the utilization of input-output relationships. The fundamental resolution for this architecture involves implementing a second network.

In pursuit of this objective, the authors opted for the Luong attention-based model. The weight, denoted as $w_t$, is computed for each timestep *t* in the source during the decoding process of the attention-based encoder-decoder, with the constraint $\Sigma _sw_t(s)=1$ and $\forall s; w_t(s) \ge 0$. The hidden state $h_t$ serves as a function representing the predicted token for the corresponding timestep, given by $\Sigma _sw_t(s) * \hat{h}_s$.

Various mathematical applications of the attention mechanism exhibit differences in how they calculate weights. In the Luong model, the computation involves applying the softmax function to the scaled scores of each token. The matrix $W_a$ linearly transforms the dot product of the decoder’s $h_t$ and the encoder’s $\hat{h}_s$ to obtain the score.

### Metaheuristics and hyperparameter optimization

Metaheuristic algorithms have many successful implementations in different areas, including wireless sensor networks^20^, hybridizing by K-means algorithm for text-document clustering^21^, tuning LSTM models^22^, convolutional neural network architecture design^23^, feature selection^24^, fraud detection^25,26^, and many others^27–29^.

In the domain of metaheuristics, hyperparameter optimization has a crucial role when tuning the operations of specific algorithms. Hyperparameter optimization is the process of selecting the right configuration of hyperparameters in a specific method for a given optimization problem. The choice of hyperparameters significantly influences the algorithm’s convergence, robustness, and overall efficacy. It is important to note that hyperparameter optimization itself is an NP-hard problem and metaheuristics are shown to be successful for tackling NP-hard optimization problems.

The NP-hardness of hyperparameter optimization arises from the large search space of possible configurations and the computational effort required to identify the optimal set of hyperparameters. In an NP-hard problem, the time required to find an optimal solution grows exponentially with the problem size, making it impractical to perform an exhaustive search. Therefore, finding the best set of hyperparameters efficiently is a formidable challenge. To tackle the NP-hard nature of hyperparameter optimization, metaheuristics offer an efficient and effective approach. Metaheuristics are a class of optimization algorithms that are designed to handle complex, large-scale problems, often characterized by non-linearity and high dimensionality.

It is important to highlight that no one-size-fits-all solution exists when it comes to optimization problems. This assertion is underpinned by the No Free Lunch (NFL)^30^ theorem, which stipulates that no universally optimal approach functions equally well for all existing problems. Consequently, the diverse field of metaheuristics has emerged, each with its own set of advantages and disadvantages. Selection is essential when determining an appropriate metaheuristic for a given problem domain, considering the problem’s characteristics and the algorithm’s strengths and weaknesses.

## Proposed method

This section presents the base Crayfish Optimization Algorithm (COA)^4^, as well as the inspiration behind the preparation of an altered version used for the purposes of our research. Subsequently, details and pseudocode of the modified algorithm are provided.

### Original crayfish optimization algorithm

The COA^4^, a novel optimization metaheuristic emulates the foraging, avoidance, and social behavior patterns observed in crayfish populations^4^. This algorithm leverages principles from the biological realm to tackle optimization problems in various fields using three distinct operating phases. These phases are designed to establish an equilibrium of exploration and exploitation. In the initial “summer resort” stage, COA focuses on exploring potential solutions. Subsequently, the “competition” and “foraging” stages simulate the exploitation phase. Transitions between these stages are influenced by temperature control. Elevated temperatures prompt crayfish to seek shelter or engage in competition for shelter, while optimal temperatures dictate foraging strategies based on food size. Temperature regulation enriches COA’s level of randomness and bolsters its global optimization capabilities.

The following equations describe the functioning of the COA:

7 $$\begin{aligned} X=\left[ X_1, X_2, \cdots , X_N\right] =\left[ \begin{array}{ccccc} X_{1,1} &{} \cdots &{} X_{1, j} &{} \cdots &{} X_{1, {\text {dim}}} \\ \vdots &{} \cdots &{} \vdots &{} \cdots &{} \vdots \\ X_{i, 1} &{} \cdots &{} X_{i, j} &{} \cdots &{} X_{i, d i m} \\ \vdots &{} \cdots &{} \vdots &{} \cdots &{} \vdots \\ X_{N, 1} &{} \cdots &{} X_{N, j} &{} \cdots &{} X_{N, {\text {dim}}} \end{array}\right] \end{aligned}$$

here *P* denotes the population, *k* the dimensionality of said problem and *N* the population limit, $X_{i,j}$ is the position of an agent in the *i* and *j* coordinate. Agents are randomly dispersed across the search space according to:

8 $$\begin{aligned} X_{i, j}=l b_j+\left( u b_j-l b_j\right) \times r a n d, \end{aligned}$$

in which *ll* represents the lower limit, *ul* the upper limit and *rnd* is sued to introduced randomness. A major influence of agent behavior is simulated temperature defined as per the following.

9 $$\begin{aligned} \text{ temp } = \text{ rand } \times 15+20 \end{aligned}$$

Once temperatures exceed 30 agents choose to locate a cooler region to vacation and resume foraging at a more appropriate temperature. Agent intake can be approximately assumed to be normally distributed and can be determined in accordance with:

10 $$\begin{aligned} p=C_1 \times \left( \frac{1}{\sqrt{2 \times \pi } \times \sigma )} \times \exp \left( -\frac{(t e m p-\mu )^2}{2 \sigma ^2}\right) \right) \end{aligned}$$

where $\mu$ denotes the optimal agent temperature, and $\sigma$ and *C* define control parameters for the given algorithm. Crayfish will fight for cave space. This is simulated by the algorithm as a random event with a 0.5 probability of occurring once tmp exceeds 30 as:

11 $$\begin{aligned} X_{i, j}^{t+1}=X_{i, j}^t-X_{z, j}^t+X_{\text{ shade } } \end{aligned}$$

with *z* denoting a random agent. Positions are therefore adjusted in accordance with other competing individual agents.

Agent positions are updated according to:

12 $$\begin{aligned} X_{i, j}^{t+1}=X_{i, j}^t+X_{\text{ food } } \times p \times (\cos (2 \times \pi \times \text{ rand } )-\sin (2 \times \pi \times \text{ rand } )) \end{aligned}$$

During the foraging phase, COA will progress towards the most effective solution, bolstering the algorithm’s ability to exploit resources and ensuring robust convergence capabilities.

### Modified crayfish optimization algorithm

While the original COA algorithm demonstrates decent performance, it is a relatively novel algorithm with a lot of room for growth. Testing conducted using CEC standard evaluation methods suggests a lack of exploration can be associated with this algorithm. The modified version attempts to tackle this deficiency by introducing two new mechanisms.

The first introduced mechanism comes from the ABC^31^ algorithm. Depleted solutions are rejected if they do not show improvement and are replaced by newly generated solutions. Given the limited number of iterations conducted in this experiment, solutions that do not improve are rejected after two iterations if no improvement is observed. This approach has been shown to boost exploration. The second mechanism introduced is quasi-reflective learning (QRL)^32^. This technique is utilized to generate new solutions further boosting exploration. Additionally, this mechanism is utilized for the initial generation of potential solutions in the initialization stages of the algorithm. Quasi-reflected component *z* of the solution of a given solution *X* is determined as:

13 $$\begin{aligned} X^{qr}_z = rand\bigg (\frac{lb_z + ub_z}{2}, x_z\bigg ) \end{aligned}$$

where *lb* and *ub* denote lower and upper bounds of the search space and *rand* denotes a random value within the given interval. The introduced algorithm is named the modified COA (MCOA). The pseudocode for the described optimizer is presented in 1.

**Algorithm 1:**
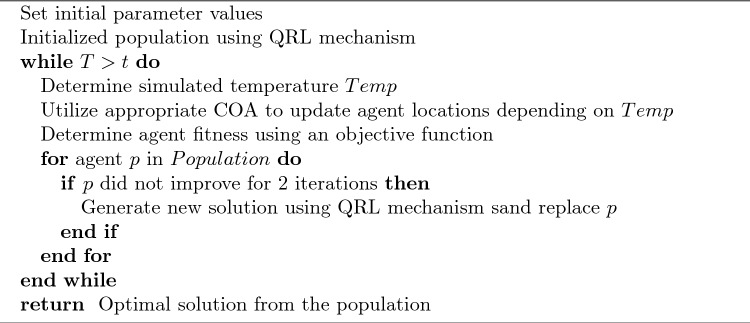
Pseudocode for the described MCOA algorithm

## Experimental setup

To establish the quality of the introduced approach, data from a publicly available clinical study is utilized^33^ that can be found on the following link https://physionet.org/content/gaitpdb/1.0.0/. The data is sourced from a collection of shoe-mounted accelerators, specifically chosen for its representation in a clinically significant study conducted by experts in the field. Moreover, the dataset is publicly available and exhibits well-organized data. One challenge associated with this dataset is its presentation in text format.

The preprocessing phase involves converting it into a suitable data frame, ensuring proper formatting, and applying labels to each patient’s sample. Patient details, including their status, are provided in a separate text file, and labels are assigned to each utilized sample based on this information. The dataset contains no missing values and all values are normalized therefore appropriate as inputs for a model. The original data is structured as a time series, and information from various patients is amalgamated to construct a balanced and unified dataset for time-series classification using the TensorFlow time series generator. The number of lags is set to 15, and a batch size of 1 is employed in the process.

Network architecture parameters including the number of layers and neurons per layer are optimized for an LSTM attention model (LSTM-ATT). Constraints for these two parameters as as follows [1, 3] layers and [5, 15] neurons per layer. Additionally, training parameters are selected. The number of training epochs, dropout, and learning rate are optimized in ranges [30, 60], [0.05, 0.2], and [0.0001, 0.01] respectively. Early stooping is also utilized to prevent overtraining with the threshold set to 1/3 of the selected number of training epochs. Respective ranges are presented in Table 1.

**Table 1:** Hyperparamaters and their respective ranges.

Hyperparameter	Lower boundary	Upper boundary
Learning rate	0.0001	0.01
Dropout	0.05	0.2
Epochs	30	60
Number of LSTM layers	1	3
Neurons in LSTM layers	5	15
Neurons in attention layer	5	15

Several metaheuristics are included in a comparative analysis of LSTM-ATT hyperparameter tuning. The introduced MCOA algorithm alongside the original COA^4^ are tested. Several well-established algorithms are included in the comparison as well such as the GA^34^, PSO^35^, FA ^36^, GWO^37^, BSO ^38^ and COLSHADE^39^ algorithm. All metaheuristics are implemented under identical testing conditions with a population size of five agents and with six allocated iterations for optimization. All metaheuristics are implemented specifically for this study with control parameter values set to those suggested in the original works. Finally, experiments are repeated 30 times to ensure a valid comparison that accounts for some of the inherent randomness in these algorithms.

To facilitate a comparison between the optimization potential of the assessed algorithms standard testing metrics including accuracy, precision, recall, and f1-score are utilized. To support the optimization process error rate is used as the objective function determined as per the:

14 $$\begin{aligned} Error\_rate = 1 - Accuracy \end{aligned}$$

An additional metric Cohen’s kappa is included as it may provide a better assessment of datasets that have an inherent imbalance. These metrics are used as the indicator function during the optimization and outcomes are logged through the entire process for each evaluated algorithm. The metrics are calculated according to:

15 $$\begin{aligned} \kappa = \frac{v_o - v_e}{1 - v_e} \end{aligned}$$

where $v_o$ denotes the observed and $v_e$ expected values.

**Figure 1:**
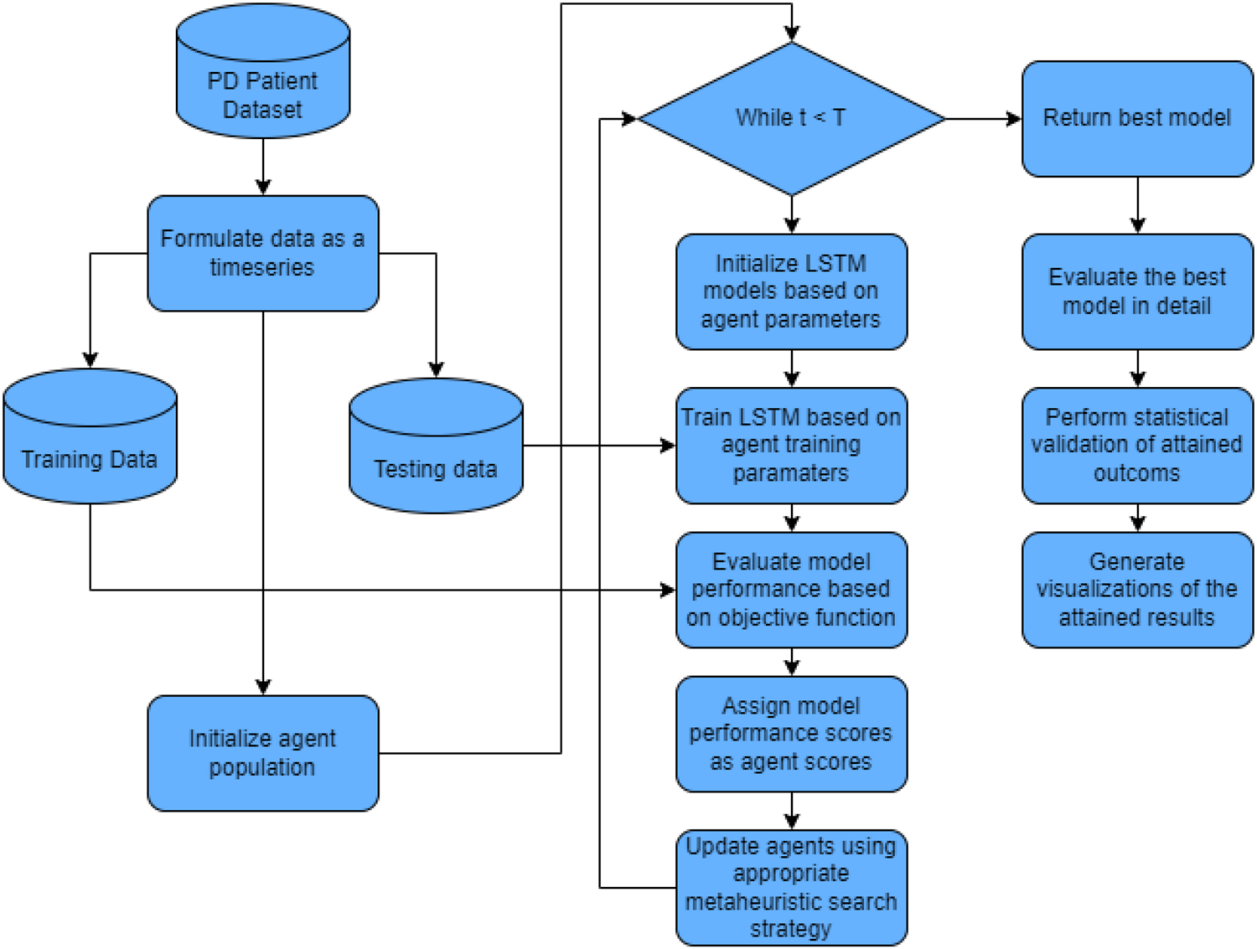
Flowchart of the proposed model evaluation process.

A flowchart of the proposed process is provided in Fig. 1.

## Simulation outcomes

Objective function outcomes during simulations in terms of best, worst as well as mean and median outcomes are provided in Table 2 and in terms of indicator function in Table 3.

**Table 2:** Overall objective function simulation outcomes.

Method	Best	Worst	Mean	Median	Std	Var
LSTM-ATT-MCOA	**0.125813**	**0.148952**	**0.134325**	**0.130152**	**0.008094**	**0.000065**
LSTM-ATT-COA	0.146059	0.200083	0.172110	0.180870	0.021438	0.000460
LSTM-ATT-GA	0.149985	0.233654	0.193699	0.198223	0.033702	0.001136
LSTM-ATT-PSO	0.134387	0.202045	0.156409	0.143167	0.025779	0.000665
LSTM-ATT-FA	0.156595	0.194815	0.172751	0.163413	0.016863	0.000284
LSTM-ATT-GWO	0.138106	0.175705	0.158599	0.165272	0.016807	0.000282
LSTM-ATT-BSO	0.129739	0.209173	0.167875	0.165995	0.027242	0.000742
LSTM-ATT-COLSHADE	0.137796	0.172606	0.161264	0.167441	0.012960	0.000168

The best metrics' values are in [bold].

**Table 3:** Overall objective function simulation outcomes.

Method	Best	Worst	Mean	Median	Std	Var
LSTM-ATT-MCOA	**0.748827**	**0.701928**	**0.731501**	**0.739508**	**0.016297**	**0.000266**
LSTM-ATT-COA	0.707837	0.601260	0.656257	0.638878	0.042468	0.001804
LSTM-ATT-GA	0.700919	0.533430	0.613713	0.605194	0.067357	0.004537
LSTM-ATT-PSO	0.731195	0.597622	0.687709	0.712931	0.050836	0.002584
LSTM-ATT-FA	0.687746	0.611343	0.655393	0.674519	0.033671	0.001134
LSTM-ATT-GWO	0.723903	0.649335	0.683318	0.670287	0.033142	0.001098
LSTM-ATT-BSO	0.741141	0.583429	0.664764	0.667567	0.054118	0.002929
LSTM-ATT-COLSHADE	0.725207	0.654987	0.678131	0.665418	0.026003	0.000676

The best metrics' values are in [bold].

**Figure 2:**
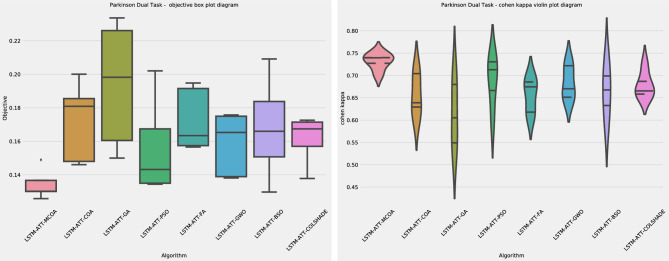
Outcome distributions for the objective and indicator function outcomes.

As can be observed in Table 2 as well as Table 3 models optimized by the introduced MCOA attained the best outcomes in terms of objective and indicator functions in all test cases. Furthermore, admirable stability has been demonstrated across all cases. Algorithm stability is further showcased in the distribution plots for the objective and indicator functions shown in Fig. 2

**Figure 3:**
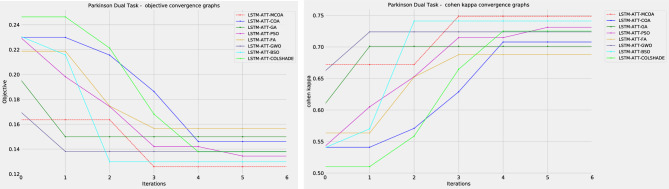
Algorithm convergence in terms of objective and indicator function outcomes.

**Figure 4:**
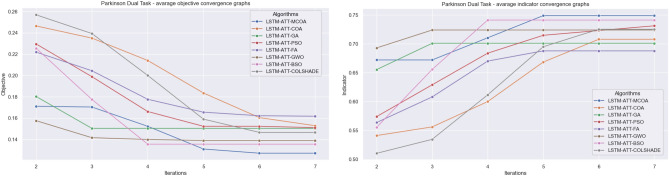
Average algorithm convergence in terms of objective and indicator function outcomes.

As shown in Fig. 2 the introduced modified metaheuristic demonstrates reliable outcomes ahead of competing algorithms. The introduced algorithm outperformed the original version of the algorithm as well as others included in the comparative analysis. Convergence rate changes in the observed algorithm can be seen in the convergence graphs in terms of objective and indicator functions in Fig. 3 and average objective and convergence graphs shown in Fig. 4.

An improvement in convergence rate can be observed for the introduced algorithm. The original COA showcases a slow convergence after stagnating at a local minimum. However, the modification introduced in this work helps the agents locate a better solution within the solution space. A detailed comparison between the best-performing models is showcased in Table 4.

**Table 4:** Detailed metric comparison between the best performing models.

Method	Metric	Control	Parkinson’s	Accuracy	Macro avg	Weighted avg
LSTM-ATT-MCOA	Precision	0.916741	0.837348	**0.874187**	**0.877045**	**0.878041**
	Recall	0.829907	0.920746	**0.874187**	**0.875327**	**0.874187**
	F1-score	**0.871166**	**0.877069**	**0.874187**	** 0.874117**	**0.874043**
LSTM-ATT-COA	Precision	0.864271	0.843484	0.853941	0.853877	0.854138
	Recall	0.848247	0.859928	0.853941	0.854087	0.853941
	F1-score	0.856184	0.851626	0.853941	0.853905	0.853962
LSTM-ATT-GA	Precision	0.911200	0.801774	0.850015	0.8564866	0.857860
	Recall	0.783757	0.919686	0.850015	0.8517215	0.850015
	F1-score	0.842687	0.856692	0.850015	0.8496893	0.849514
LSTM-ATT-PSO	Precision	0.876414	**0.854711**	0.865613	0.865563	0.865835
	Recall	**0.858928**	0.872643	0.865613	0.865785	0.865613
	F1-score	0.867583	0.863584	0.865613	0.865583	0.865634
LSTM-ATT-FA	Precision	0.904270	0.795534	0.843405	0.849902	0.851267
	Recall	0.776703	0.913541	0.843405	0.845122	0.843405
	F1-score	0.835646	0.850464	0.843405	0.843055	0.842869
LSTM-ATT-GWO	Precision	0.880697	0.843699	0.861894	0.862198	0.862663
	Recall	0.845022	0.879636	0.861894	0.862329	0.861894
	F1-score	0.862491	0.861293	0.861894	0.861892	0.861907
LSTM-ATT-BSO	Precision	**0.923059**	0.826636	0.870261	0.874848	0.876058
	Recall	0.814792	**0.928587**	0.870261	0.871689	0.870261
	F1-score	0.865553	0.874651	0.870261	0.870102	0.869988
LSTM-ATT-COLSHADE	Precision	0.923832	0.813368	0.862204	0.868600	0.869986
	Recall	0.796856	0.930918	0.862204	0.863887	0.862204
	F1-score	0.855659	0.868182	0.862204	0.861920	0.861763
	Support	4962	4719			

The best metrics' values are in [bold].

**Figure 5:**
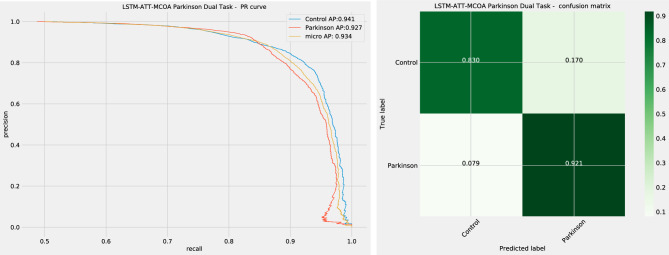
Best performing model PR plot and confusion matrix.

As shown in Table 4 the introduced algorithms demonstrate the highest accuracy and a high f1-score for both PD and control group identification. However, admirable results are shown by the PSO and BSO algorithms in terms of PD and control group when observing precision alone. These outcomes are to be expected as per the NFL, no single approach will work equally well across all metrics and test cases. Further details of the best-performing model are shown in Fig. 5.

Finally, to facilitate experimental repeatability, the hyperparameter choices made by optimizers for the best-performing models are presented in Table 5.

**Table 5:** Hyperparameter choices made for best-performing models constructed by optimizers.

Method	Learning rate	Dropout	Epochs	Layers	Neurons L1	Neurons L2
LSTM-ATT-MCOA	0.010000	0.050000	60	1	15	N/a
LSTM-ATT-COA	0.010000	0.059956	60	2	14	10
LSTM-ATT-GA	0.010000	0.180735	60	1	15	N/a
LSTM-ATT-PSO	0.010000	0.052466	60	1	15	N/a
LSTM-ATT-FA	0.009148	0.153722	44	1	12	N/a
LSTM-ATT-GWO	0.010000	0.200000	60	2	15	15
LSTM-ATT-BSO	0.010000	0.200000	60	1	15	N/a
LSTM-ATT-COLSHADE	0.008276	0.200000	60	1	15	N/a

### Outcome statistical validation

Within the realm of optimization problems, the assessment of models emerges as a crucial focal point. Understanding the statistical significance of implemented enhancements becomes imperative, as a reliance solely on outcomes falls short of establishing the superiority of one algorithm over another.

According to prior investigations^40^, a judicious statistical assessment should transpire only subsequent to the thorough sampling of the evaluated methods. This involves the establishment of objective averages across numerous independent runs, with an additional prerequisite that the samples adhere to a normal distribution to preclude erroneous conclusions. The utilization of objective function averages remains an unresolved inquiry in the comparison of stochastic methods among researchers^41^.

In order to establish the statistical significance of the observed results, the optimal values from 30 independent executions of each metaheuristic were employed to construct the samples. However, the judicious application of parametric tests necessitated verification. To this end, compliance with the recommendations of^42^ was ensured, encompassing considerations of independence, normality, and homoscedasticity of data variances.

**Figure 6:**
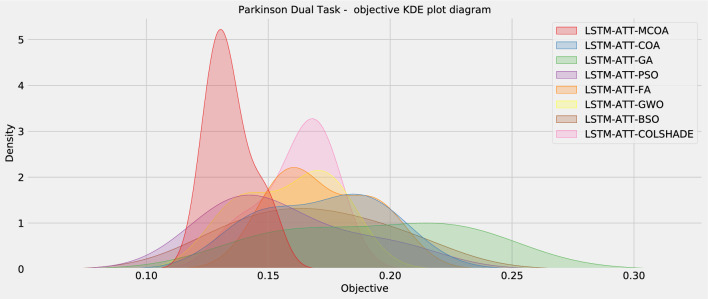
Objective function KDE plot.

The independence criterion is met by virtue of initializing each run with a pseudo-random number seed. Nevertheless, the normality condition remains unmet, as evidenced by KED plots shown in Fig. 6 and substantiated by Shapiro-Wilk test outcomes for single-problem instance analysts^43^. By performing the Shapiro-Wilk test, *p*-values are generated for each method-problem combination, and these outcomes are presented in Table 6.

**Table 6:** Shapiro-Wilk scores for the single-problem analysis for testing normality condition.

Experiment	MCOA	COA	GA	PSO	FA	GWO	BSO	COLSHADE
PD LSTM-ATT	0.035	0.032	0.022	0.027	0.027	0.031	0.018	0.012

The conventional significance levels represented by $\alpha = 0.05$ and $\alpha = 0.1$ indicate the potential rejection of the null hypothesis ($H_0$). This implies that none of the samples, spanning diverse problem-method combinations, adhere to a normal distribution. These findings signal the failure to meet the normality assumption, a prerequisite for the robust application of parametric tests. Consequently, the verification of homogeneity of variances was considered unnecessary.

Given the unmet prerequisites for the reliable use of parametric tests, non-parametric tests were employed for subsequent statistical analyses. Specifically, the Wilcoxon signed-rank test, acknowledged as a non-parametric statistical method^44^, was conducted on the MCOA method and all alternative techniques in the conducted experiment. The same data samples utilized in the preceding normality test (Shapiro-Wilk) were applied for each method. The outcomes of this analysis are detailed in Table 7.

**Table 7:** Wilcoxon signed-rank test findings.

MCOA versus others	COA	GA	PSO	FA	GWO	BSO	COLSHADE
PD LSTM-ATT	0.040	0.044	0.045	0.033	0.025	0.034	0.035

Table 7, which presents the *p*-values obtained from the Wilcoxon signed-rank test, demonstrates that when tackling LSTM-ATT optimization the proposed MCOA method achieved significantly better performance than all other techniques in all three experiments.

The *p*-values for all other methods were lower than 0.05. Therefore, the MCOA technique exhibited both robustness and effectiveness as an optimizer in these computationally intensive simulations. Based on the statistical analysis, it can be concluded that the MCOA method outperformed most of the other metaheuristics investigated in all four experiments.

## Conclusion

This work tackles PD detection from patient gate data collected from a show-mounted accelerometer sensor as a noninvasive way for early diagnosis. Timely treatments are crucial for battling this neurodegenerative disease as there is currently no way of undoing the damage caused by the condition. This task is tackled through the application of AI algorithms. Attention-based LSTM models are trained on real-world data, and asses on their ability to detect signs of the condition. Furthermore, an altered variation of a relatively novel algorithm is proposed and applied to hyperparameter tuning to improve model performance. The introduced approach has shown admirable outcomes with the best-constructed models exceeding 87% accuracy. Meticulous statistical validations confirmed the observations and enforced that the introduced MCOA outperformed the original algorithm when applied to hyperparameter optimization of LSTM-ATT networks as well as competing optimizers in a statistically significant way.

Like any research, this study is not without its limitations. The inclusion of optimization algorithms in the comparative analysis has been restricted due to computational constraints. Similarly, the optimization process is constrained by the use of limited model population sizes. The potential for improved outcomes exists with the allocation of additional resources. Moreover, the current testing is based on the limited available data samples from dual-task walking tests with accelerometers, as only a restricted amount of data is presently accessible for Parkinson’s disease diagnosis.

Future research aims to refine early detection methods and explore other contemporary recurrent networks for addressing the task at hand. The introduced optimization algorithm will also be investigated for potential applications in computer security and hyperparameter optimization.

## Data Availability

The datasets used and analysed during the current study is freely available from the following URL: https://physionet.org/content/gaitpdb/1.0.0/.
